# The Enhanced Pharmacological Effects of Modified Traditional Chinese Medicine in Attenuation of Atherosclerosis Is Driven by Modulation of Gut Microbiota

**DOI:** 10.3389/fphar.2020.546589

**Published:** 2020-10-15

**Authors:** Wenyan Ji, Ting Jiang, Zheng Sun, Fei Teng, Chenchen Ma, Shi Huang, Suhua Yan

**Affiliations:** ^1^ School of Medicine, Shandong University, Jinan, China; ^2^ Department of Cardiology, Qingdao Municipal Hospital of Traditional Chinese Medicine (Qingdao Hiser Medical Group), Qingdao, China; ^3^ Single-Cell Center and Shandong Key Laboratory of Energy Genetics, Qingdao Institute of BioEnergy and Bioprocess Technology, Chinese Academy of Sciences, Qingdao, China; ^4^ College of Food Science and Engineering, Hainan University, Haikou, China; ^5^ Department of Cardiology, Qianfoshan Hospital of Shandong Province, School of Medicine, Shandong University, Jinan, China

**Keywords:** traditional Chinese medicine (TCM), atherosclerosis, gut microbiota, cardiovascular disease, combination of Chinese and Western medicine

## Abstract

Accumulating evidence indicated that gut microbiota-targeted therapy is a promising strategy to treat Cardiovascular Disease (CVD). Traditional Chinese Medicine (TCM) has been used in CVD treatments for over 2,000 years which is believed to result from the modulation of gut microbiota, yet the underlying mechanism remains elusive. According to the theoretical system of TCM, we developed an innovative formula of TCM named “TongMai ZhuYu (TMZY)” on top of one classic Chinese herbal formula [“XueFu ZhuYu (XFZY)”], which can more effectively alleviate CVD in the clinical practice. Here, we first systematically assessed the pharmacological effects of TMZY, XFZY, and atorvastatin on atherosclerosis (AS) induced by high-fat diet (HFD) in rats. TMZY typically outperformed others in alleviating AS rats by characterization of pathological morphology, immunohistochemistry, inflammatory cytokines. Remarkably, combining this modified TCM formula (TMZY) with atorvastatin can further help the alleviation of AS in rats by suppressing immune and inflammatory responses. Furthermore, to test whether TMZY alleviated AS symptoms by altering gut microbial compositions (dysbiosis), we employed 16S amplicon sequencing to investigate gut microbiota changes in the AS mice induced by high choline diet (HCD) using both TMZY and XFZY under antibiotic-treated and untreated conditions. TCM formulas induced consistent and remarkable changes in the phenotypes and microbiota in the HCD mice. TMZY modulated more changes in the gut microbiota to improve diseased phenotypes than XFZY. Notably, the TMZY-intervention effect on CVD in mice attenuated after the suppression of gut microbial activity by antibiotics. Collectively, we demonstrated that TCM herbals could effectively modulate the gut microbiota as a mechanism for altering the pathogenesis of cardiovascular disorders in mice/rats.

## Introduction

Cardiovascular Diseases (CVD) are the number one cause of global death (around 31% annually in 2016). Atherosclerosis (AS) is widely considered as the most common issue for the prevention and treatment of CVD (Benjamin et al., 2018). The typical treatments (such as statins) in Western medicine against AS exhibited a substantial effect on anti-AS and lowering lipid. However, the side effects, such as gastrointestinal symptoms, bleeding risk, damage of liver and kidney function, were commonly reported and might limit their applications in clinical practice (Stone et al., 2015). Hence, clinicians have begun to consider the potential role of Traditional Chinese Medicine (TCM) in the prevention and treatment of CVD because of the unmet needs for CVD control using Western medicine. Multiple basic and clinical studies related to TCM formulas have drawn increasing attention from the cardiovascular community (Hao et al., 2017; Wang et al., 2018). In TCM, blood “stasis” is widely considered as a key pathogenic factor targeted to treat CVD and especially AS (Chen, 2015; Xu and Chen, 2017). Multiple TCM formulas or Chinese herbal monomers have proven to be active on the regulation of serum lipid metabolism, protection of vascular endothelial cells, anti-coagulation, anti-oxidation, inhibition of inflammation, *etc.* (Liu et al., 2012; Liu and Huang, 2016; Zhang et al., 2019).

The TCM herbal formulas are still keeping updated in the modern medical practice of CVD control. For example, the standardized Xuefu Zhuyu decoction (XFZY) has been a treatment of blood stasis (AS) since the 19th century in China (Yang et al., 2017; Lin et al., 2018; Meng et al., 2018). To reinforce the therapeutic effects of XFZY, we creatively added in new compounds into XFZY and further formed a new formula termed TongMai ZhuYu decoction (TMZY), which has been reported to exhibit significant clinical efficacy on AS (Ji et al., 2012; Ji et al., 2017). According to the modern pharmacological research, newly added compounds play vital roles in anti-inflammation and lipid lowing, such as *Citrus aurantium* L. (Zhi Shi) (Li and Wang, 2011), *Crataegus pinnatifida* Bunge (Shan Zha) (Fan et al., 2006; Akila and Devaraj, 2008), *Reynoutria multiflora* (Thunb.) Moldenke (syn. *Fallopia multiflora* (Thunb.) Harald.) (Shou Wu) (Zhang et al., 2018), and *Alisma Plantago-aquatica* Linn. (Ze Xie) (Zhang et al., 2017). However, the unsolved questions here include: (1) Does our updated TCM formula outperform conventional TCM formula and Western medicine in the attenuation of AS? And why? (2) Can the synergy of the new TCM formula and Western medicine further improve the therapeutic potentials significantly? And why?

It is well known that the gut microbiome plays a vital role in metabolic and cardiovascular-related disorders (Marchesi et al., 2016). Even though a dysbiotic gut microbiome has actively associated with AS (Koren et al., 2011; Jie et al., 2017; Jonsson and Backhed, 2017; Komaroff, 2018), which is supported by increasing mechanistic evidence, but it is still not clear whether CVD or AS can be successfully treated by targeting the gut microbiota. Intriguingly, TCM has been serving for therapeutic and prophylactic management of multiple metabolic diseases for centuries, while, recently, their clinical efficacy has proven to be greatly attributed to structural or functional modulation of gut microbiota (Xu et al., 2015; Xu et al., 2017; Tong et al., 2018; Wei et al., 2018; Wu et al., 2019). There are still a considerable number of herbal formulas that have been widely used in clinical application and displayed satisfying efficacy for hundreds of years, yet their links to gut microbiota remain mostly unknown. Therefore, to examine the involvement of the gut microbiome in the alleviation of CVD would be of both clinical and scientific significance for the inheritance and innovation of TCM decoctions.

To answer those questions above, firstly, we developed the AS rat model, and then systematically examined *i*) the clinical efficiency of a novel TCM formula on AS; *ii*) the potential synergistic effects of the innovative TCM formula and Western medicine on treating AS, and *iii*) elucidated if the structural modulation of gut microbiome may causatively contribute to the significant effect of modified TCM formula exerted on AS.

## Materials and Methods

### Animals

Specific-pathogen-free (SPF) male Wistar rats (weighting 200 ± 10 g) were purchased from Qingdao Peitefude rat breeding Co., Ltd. (Qingdao, China, license number: SCXK(Lu)20130004). Eight-week-old male and female C57BL/6J mice (SPF, 22–25 g) were obtained from Beijing Huafukang Bioscience Co., Ltd. (Beijing, China, license number: SCXK(Jing)20140004). Animals were housed in cages under standardized conditions: a 12-h light/dark cycle, 23 ± 2°C room temperature, and 55 ± 5% relative humidity. Animals had free access to food and water. The Ethics Committee of Qingdao University approved all the experimental procedures, and animals were treated according to the recommendations in the Guide for the Care and Use of Laboratory Animals of the National Institutes of Health. The ethical committee approval number of this project is 2017HC11LQ076.

### Additives and Drugs

HFD chow contained 1% cholesterol, 0.5% sodium cholate, 0.2% propylthiouracil, 5% lard oil, and 93.3% common feed (Teluofei Experimental Animal Feeding Material Technology Co., Nantong, China). HCD was also prepared by Teluofei Experimental Animal Feeding Material Technology Co.

The formulations of XFZY and TMZY were shown in **Tables 1**, **2**. The Chinese medicine herbs were made into decoction-free granules by Beijing Kangrentang Pharmaceutical Co., Ltd. (Beijing, China). Granules were dissolved in ultrapure water, and the suspension was stored at 4°C.

**Table 1:** Composition of Xuefu Zhuyu decoction (XFZY, one dose).

Herb	Family	Part used	Amount used (g)	Batch number
**Peach kernel (*Prunus persica (L.) Batsch.*)**	Rosaceae	Seed	12	17004701
**Flos carthami (*Carthamus tinctorius L.*)**	Compositae	Flower	9	17009732
**Angelica sinensis (*Angelica sinensis (Oliv.) Diels*)**	Umbelliferae	Root	9	17013382
**Radix rehmanniae (*Rehmannia glutinosa (Libosch.) DC.*)**	Scrophulariaceae	Root	9	17012251
**Rhizoma Chuanxiong (*Ligusticum anthriscoides* ‘Chuanxiong’)**	Umbelliferae	Root	4.5	17012231
**Radix paeoniae rubra (*Paeonia lactiflora Pall.*) Paeonia lactiflora Pall.**	Ranunculaceae	Root	6	17003771
**Achyranthesbidentata (*Cyathula officinalis K.C.Kuan*)**	Amaranthaceae	Root	9	17009561
**Platycodon grandiflorum (*Platycodon grandiflorus (Jacq.) A. DC.*)**	Platycodon	Root	4.5	17007341
**Radix Bupleuri (*Bupleurum chinensis DC.*)**	Umbelliferae	Root	3	17010941
**Fructus Aurantii (*Poncirus trifoliata (L.)Raf.*)**	Rutaceae	Fruit	6	17010052
**Radix liquiritiae (*Glycyrrhiza uralensis Fisch.*)**	Leguminosae	Root	3	17014772

**Table 2:** Composition of Tongmai Zhuyu decoction (TMZY) (one dose).

Herb	Family	Part used	Amount used (g)	Batch number
**Radix rehmanniae (*Rehmannia glutinosa (Gaertn.) DC.*)**	Scrophulariaceae	Root	9	17012251
**Peach kernel (*Prunus persica (L.) Batsch.*)**	Rosaceae	Seed	12	17004701
**Flos carthami (*Carthamus tinctorius L.*)**	Compositae	Flower	9	17009732
**Radix paeoniae rubra (*Paeonia lactiflora Pall.*)**	Ranunculaceae	Root	6	17003771
**Rhizoma Chuanxiong (*Ligusticum anthriscoides ‘Chuanxiong’*)**	Umbelliferae	Root	4.5	17012231
**Angelica sinensis (*Angelica sinensis (Oliv.) Diels*)**	Umbelliferae	Root	9	17013382
**Achyranthesbidentata (*Cyathula officinalis K.C. Kuan*)**	Amaranthaceae	Root	9	17009561
**Hawthorn (*Crataegus pinnatifida Bge.*)**	Rosaceae	Fruit	6	17006711
**Citrus aurantium L. (*Citrus junos Sieb. ex Tanaka*)**	Rutaceae	Fruit	6	17012131
**Rhizoma alismatis (*Alisma plantago-aquatica subsp. orientale (Sam.) Sam.*)**	Alismaceae	Tuber	9	17002371
**Polygonum multiflorum (*Reynoutria multiflora (Thunb.) Moldenke*)**	Polygonaceae	Root	9	16028042

Atorvastatin calcium (Lipitor^®^), Vitamin D3 for injection and propylthiouracil were purchased from Pfizer (New York, NY, USA), Shanghai general pharmaceutical Company (Shanghai, China), and Shanghai Zhaohui Pharmaceutical Co., Ltd. (Shanghai, China), respectively. We obtained quadruple antibiotic including neomycin, vancomycin, ampicillin, and metronidazole from Qingdao Jieshikang Biotechnology Co., Ltd. (Qingdao, China).

### Animal Grouping and Model Construction

A total of 48 rats were randomly assigned into six groups (*n* = 8 for each) using random number table method: the healthy control group, HFD-induced group, atorvastatin-treated group, XFZY-treated group, TMZY-treated group, and integrative group. Rats in the healthy control group were fed with common diet, and other rats were fed on HFD with the administration of vitamin D3 by injection (175,000 IU/kg, once per 2 days, four times totally).

A total of 42 C57BL/6J mice were divided into seven groups (*n* = 6 for each) using random number table method: the control group, high-choline group, choline + XFZY group, choline + TMZY group, choline + antibiotic group, choline + antibiotic + XFZY group, and choline + antibiotic + TMZY group. Mice in the control group were fed with common diet, and other mice were fed with high-choline chow.

### HPLC Analysis of TMZY Extracts

**Figure 1:**
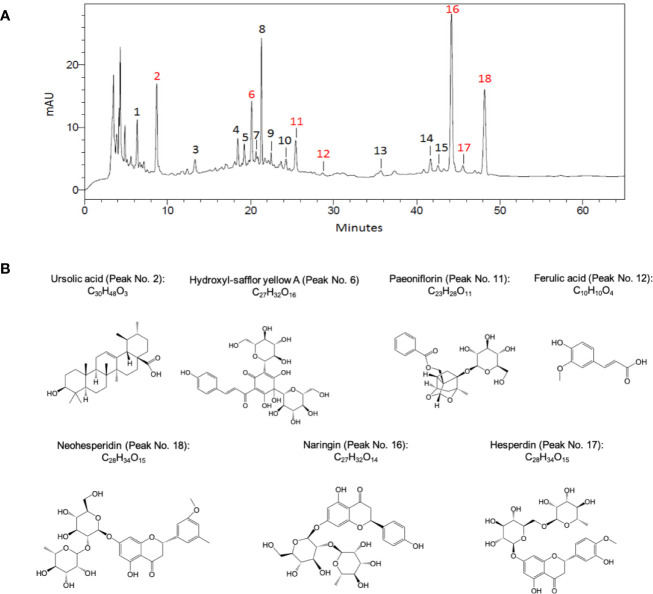
The chromatographic profile of TMZY extracts and chemical structures of major pharmaco-active compounds identified. **(A)** Typical high-performance liquid chromatography (HPLC) chromatograms of TMZY extracts. mAU, milli-absorbance unit. **(B)** Chemical structures of the major pharmaco-active compounds identified. Peak No.: 2. Ursolic acid; 6. Hydroxyl-safflor yellow A; 11. Paeoniflorin; 12. Ferulic acid; 16. Naringin; 17. Hesperdin; 18. Neohesperidin.

The extraction process of TMZY extracts was conducted as previously reported. Briefly, one dose of TMZY medicines were added in 10 volumes of water, soaked for 60 min, and decocted two times (3 h at the first time and 2 h at the second time). The two decoctions were then combined together. In order to control the quality of extracts, the fingerprint spectrum was measured by a high-performance liquid chromatography (HPLC) method, and the chromatographic profile of the TMZY extracts is presented in **Figure 1**. The analyses were conducted by Shimadzu system (LC-20AT, Shimadzu Corp., Japan) equipped with an auto-sampler, a quaternary gradient pump, and a SPD-20A detector. The mobile phase was acetonitrile (A) – 0.2% formic acid solution (B). The mobile phase gradient elution program is shown in **Table 1** and the flow rate was 1.0 ml/min. The chromatographic column was Shim-pack VP-ODS C18 (4.6 mm × 250 mm, 5 μm) with working temperature of 30°C. The detector wavelength was set at 254 nm. The peak assignment in **Figure 1** is listed in **Table S1**.

### Drug Administration

For Wistar rats, atorvastatin (2 mg/kg per day), XFZY (1.152 g/kg per day), and TMZY (1.224 g/kg per day) were given by gavage. Rats in the healthy control and HFD-induced groups received an equal volume of normal saline by gavage. Oral gavage at 9 am daily was performed for 8 weeks, and rats were weighed once a week.

For C57BL/6J mice, XFZY (1.664 g/kg per day), TMZY (83.5 mg/kg per day), and distilled water were given by gavage at 9 am, and the volume of XFZY, TMZY, or distilled water was 0.5 ml. Solution of quadruple antibiotic consisted of neomycin (100 mg/kg), vancomycin (50 mg/kg), ampicillin (10 mg/kg), and metronidazole (100 mg/kg). Mice were administered with quadruple antibiotic (10 ml/kg) by gavage 12 h^-1^ for 4 weeks (Reikvam et al., 2011). The fresh antibiotic concoction was mixed every day, and ampicillin and distilled water were renewed every 7th day.

### Examination of Blood Lipid and Histopathological Observation

Eight weeks later, rats were anesthetized with 10% chloral hydrate (4.5 ml/kg) intraperitoneally after being fasted for 12 h, and rat blood and aortas were collected. Serum was separated to measure the concentration of blood lipid (TC, TG, LDL-C, HDL-C) using an automatic biochemical analyzer. The aortas were rinsed in normal saline and fixed in 10% formalin. Then, 5 mm thoracic aorta was dehydrated, paraffin embedded, and cut into slices (5 μm). Afterward, the slices were hematoxylin-eosin (HE) stained, followed by histopathological observation under an optical microscope (BX43, Olympus, Tokyo, Japan). The thickness of intima and media was analyzed by using the ipp6.0 software.

### Expression of TGF-β, VCAM-1, HMGB-1, and FOXP3 by IHC

After dewaxing and hydration using 3% hydrogen peroxide, slices of thoracic aorta were incubated with rabbit polyclonal to TGF-β antibody (ab92486, Abcam, Cambridge, UK, 1:100), goat polyclonal to VCAM-1 antibody (sc-1504, Santa Cruz Biotechnology, Inc., Santa Cruz, CA, USA, 1:25), rabbit polyclonal to HMGB-1 antibody (ab18256, Abcam, 1:50), or mouse monoclonal to FOXP3 antibody (ab22510, Abcam, 1:50) at 4°C overnight. Then, slices were incubated with horseradish peroxidase (HRP)-conjugated secondary antibodies such as goat anti-rabbit antibody (ab6721, Abcam), goat anti-mouse antibody (ab6789, Abcam), and rabbit anti-goat antibody (ab6721, Abcam) at 37°C for 60 min. Immunopositive reaction was visualized by with a 3,3’-diaminobenzidine (DAB) chromogenic reagent kit (Beijing ZhongshanJinqiao Biotechnology Co., Ltd., Beijing, China). The slices were imaged with a light microscope.

Immunostaining was assessed according to immunoreactive score (IRS) on the basis of a semiquantitative approach. IRS = staining intensity (SI) × percentage of positive cells (PP). The staining intensity (SI) was scored as follows: 0 (no staining), 1 (light yellow), 2 (dark yellow or light brown), and 3 (dark brown). Positive cells were counted at a magnification of ×200 in three independent fields. PP was defined as follows: 0 (0–5% positive cells), 1 (6–25% positive cells), 2 (26–50% positive cells), 3 (51–75% positive cells), and 4 (76–100% positive cells).

### Levels of IL-4, IFN-γ and hs-CRP by Enzyme-Linked Immunosorbent Assay (ELISA)

Levels of IL-4, IFN-γ and hs-CRP in the serum from rats were measured by Rat IL-4 Quantikine ELISA Kit (R4000, R&D Systems, Minneapolis, MN, USA), Rat IFN-gamma Quantikine ELISA Kit (SRIF00, R&D Systems), and Rat hs-CRP ELISA Kit (ml003217, Shanghai Enzyme-linked Biotechnology Co., Ltd., Shanghai, China), respectively, as suggested by the manufacturers.

### Measurement of the Blood TMAO Level by UPLC-MS

Four weeks later, the blood of mice was taken from the eyeballs after being fasted for 12 h. Serum was separated and stored at −80°C. We determined serum TMAO level according to a previous reference with modifications (Romano et al., 2018). In brief, d9-TMAO (Sigma-Aldrich, St. Louis, MO, USA), as an internal standard, was dissolved in methanol to obtain a 10 mM stock solution. Then, 160 μl d9-TMAO solution was mixed with 40 μl serum in a 1.5 ml EP tube, followed by vortex for 1 min and centrifugation at 13,000 × g at 4°C for 20 min. The supernatant was collected into a chromatographic bottle. Identification and quantitation of TMAO were performed using an Agilent 1290-6430 ultra-HPLC coupled to a triple quadrupole Tandem Mass Spectrometer (Agilent Technologies, Palo Alto, CA, USA) in positive multiple-reaction monitoring (MRM) mode. Liquid chromatography separation was achieved on a Luna silica column (250 mm × 4.6 mm, 5 μm particle size, Phenomenex, Torrance, CA, USA) at a flow rate of 0.7 ml/min. The mobile phase consisted of 0.1% propionic acid in water (A) and 0.1% acetic acid in methanol (B) (Wang et al., 2014). Samples were eluted as follows: 0–10 min, linear gradient 2–15% B; 10–15 min, linear gradient 15–100% B; 15–19 min, isocratic 100% B; 19–20 min, linear gradient 100–2%; 20–29 min, isocratic 2% B. The vaporizer temperature was 360°C. The concentrations of the calibration standards were 0.01–200 μM TMAO.

### The Platelet Aggregation Rate Measured by Flow Cytometry (FCM)

Whole blood (40 μl) was added into 40 μl acidic citric acid glucose (ACD) solution B and 80 μl HEPES’/tyrode buffer. After mixing, the mixture was equally distributed to EP tubes (containing 5 μl cell staining buffer) labeled E1, E2, E3, and E4, respectively. Then, 0.24 μl LCD61-PE was added to E1 and E3 tubes, respectively, while 0.24 μl CD61-FITC was added to E2 and E4 tubes, respectively. After avoiding light, the contents of E1 and E2 tubes were moved to T1 tube with a micro-pipette, while the contents in E3 and E4 tubes were transferred to T2 tube. After that, 0.8 μl 0.9% saline was added to T1 tube, and 0.8 μl ADP (concentration 20 μmol/L) was added to T2 tube. Subsequently, T1 and T2 tubes were shaken well in Vortex at 37°C for 10 min (800 rpm) to activate platelets. Then, 500 μl erythrocyte lysate was added into T1 and T2 tubes and incubated at room temperature for 10 min before tests. During analyzing, two-color fluorescent dyes of FITC and PE produced fluorescence at 525 and 572 nm, respectively, after stimulated at 488 nm. Fluorescence signals were collected by FL1 and FL2 channels of Cytomics FC500 flow cytometer, respectively. Platelets were delineated using forward scattered light (FSC) and side scattered light (SSC) two-dimensional scattering points. The scatter plots of CD61-FITC as abscissa and CD61-PE as ordinate were established. At last, the platelet aggregation rate was obtained based on the calculation of the ratio of the double positive cell groups (CD61-FITC and CD61-PE) to total platelets.

Statistical analysis on the measurements above was performed using Graphpad Prism 5 software (GraphPad, San Diego, CA, USA). The *P*-values were calculated using the one-way analysis of variance (ANOVA) or Student’s *t*-test. A *P* < 0.05 was considered a significant difference.

### Fecal Sample Collection and 16S rRNA Gene Sequencing

Feces in the caecum of mice were collected and genomic DNA was extracted from bacterial fecal pellets using a DNeasy Blood and Tissue Kit (Qiagen, Valencia, CA, USA) according to the manufacturer’s instructions. The DNA purification kit (Omega Biotech, Doraville, GA, USA) was used to remove contaminants. Primer pair 27F-534R for V1-V3 regions of bacterial 16S rRNA genes was synthesized by Beijing Genomics Institute (BGI; Shenzhen, China). PCR amplification was performed using the 2X Taq PCR Master Mix (Biomiga, San Diego, CA, USA) following the protocol developed by the Human Microbiome Project (Methe et al., 2012).

All sequences were pre-processed (sorted by barcodes and trimmed by base quality) following the standard QIIME (v.1.9) pipeline. A total of 643,038 high-quality partial 16S rRNA sequences (sequences with a read length >200 bp, a quality score >25, and no barcode errors and ambiguous bases) were obtained, with an average of 8,669 sequences per sample. The read depth was subsequently rarefied to 2,459 sequences per sample, which allowed all samples to be compared at an equivalent sequencing depth. Downstream bioinformatics analysis was performed using Parallel-Meta 3 (Jing et al., 2017), a software package for comprehensive taxonomical and functional comparison of microbial communities. Clustering (of operational taxonomic units, OTUs) was conducted at the 97% similarity level using a pre-clustered version of the GreenGenes database (Mcdonald et al., 2012). Alpha-diversity was calculated by Shannon Index and distance matrices between samples were computed based on the weighted Meta-Storms algorithm (Su et al., 2012). Beta-diversity, which indicates the diversity between different samples in a microbial community, was carried out using PCoA. The other statistical analysis, e.g., Kruskal-Wallis test, Wilcoxon rank sum test, and permutational multivariate analysis of variance (PERMANOVA), were performed *via* the R scripts integrated in Parallel-Meta 3. The rarefaction analysis and Shannon diversity index were used to estimate the richness and diversity of species. The relative abundance of differential taxonomic groups was visualized by “pheatmap” in the “pheatmap” R package. Differences in the relative abundance of taxonomic groups at the genus level between samples were evaluated with Wilcoxon rank sum test. False-discovery rate (FDR) values were estimated using the Benjamini-Hochberg method to control for multiple testing. *P* values less than 0.05 were considered statistically significant.

## Results

### Systematic Assessment of Pharmacological Effects in Atherosclerotic Rats

The HFD was used to establish the AS model in rats. The atherosclerotic rats in different groups were administrated by XFZY, TMZY (**Figure 1**), atorvastatin, and integrative treatment, respectively. To systematically assess the pharmacological effects of those treatments, we carefully examined the changes of pathological morphology, immunohistochemistry (IHC), and the levels of inflammatory cytokines.

### The Interventional Effect of TMZY on Atherosclerotic Rats

**Figure 2:**
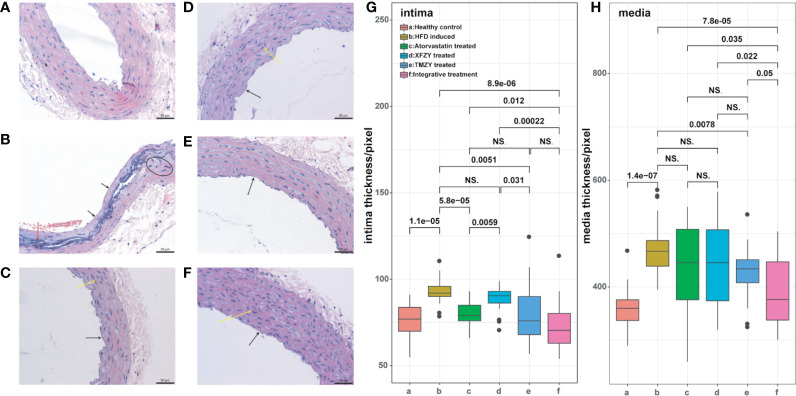
HE staining of thoracic aortic reveals the interventional effect of TCM treatments on atherosclerotic rats. **(A)** HE staining of rat thoracic aorta in healthy control group. **(B)** HE staining of rat thoracic aorta in HFD group. **(C)** HE staining of rat thoracic aorta with HFD and atorvastatin intervention. **(D)** HE staining of rat thoracic aorta with HFD and XFZY intervention. **(E)** HE staining of rat thoracic aorta with HFD and TMZY intervention. **(F)** HE staining of rat thoracic aorta with HFD and the integrative treatment. We marked mild hyperplasia of vascular SMCs with arrows. **(G–H)** The boxplots of digitalized intima-media thickness of thoracic aorta (which were generated by ipp6.0) of rats from six treatment groups. The p values of significant associations are all marked on top of boxplots, while NS donates “non-significance”.

We first found that TMZY effectively alleviated the AS-associated pathological morphology of rat thoracic aorta. HE staining of rat thoracic aorta was used to evaluate the changes of pathological morphology. Rats were fed on HFD and vitamin D3 for 8 weeks to construct AS model. Compared with the healthy control group (**Figure 2A**), aortic intima thickening, partial loss of endothelial cells, the proliferation of smooth muscle cells (SMCs), and appearance of lipid plaques, foam cell accumulation, and necrotic (deep-dyeing) substances were observed in the HFD group (**Figure 2B**).

Moreover, partial media cells in the HFD group showed a stable nucleus and necrosis, along with the disorder of the three-layers structure of the arterial wall. Those changes indicated that AS model was constructed successfully. The pathological morphology changes of rat thoracic aorta showed varying degrees of mitigation in the XFZY (conventional TCM formula) group and atorvastatin (traditional Western medicine) group. The partial areas of arterial intima were unsmooth, endothelial cells were partially exfoliated, while mild hyperplasia of vascular SMCs was observed (**Figures 2C, D** **)**. Similarly, the pathological morphology changes were alleviated effectively after TMZY intervention (**Figures 2E, F** **)**.

Moreover, attenuation of aortic intima thickening and foam cell accumulation, as well as more explicit boundaries between layers, were observed in the TMZY group compared with the XFZY group. The effect of TMZY on pathological morphology changes was similar to that of atorvastatin. Analysis of the aortic intima and media thickness using Ipp6.0 software showed varying degrees of mitigation on the thickness of intima (*P =* 0.0051) and media (*P =* 0.0078) in the TMZY group under the HFD (**Figures 2G, H** **)**. The effects of TMZY on reducing intima thickness (*P =* 0.031) were more potent than that of XFZY, while no difference in reducing media thickness between TMZY and XFZY was observed (*P =* 0.55). Also, no difference between the TMZY-treated and atorvastatin-treated groups was observed in reducing the thickness of intima and media (*P =* 0.5 and *P =* 0.58, respectively).

**Figure 3:**
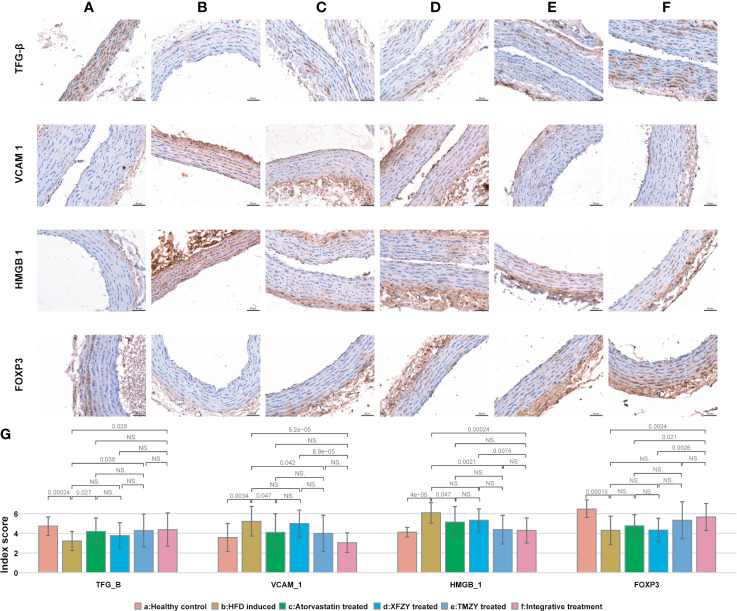
The expression of immune factors in thoracic aortic is associated with TCM treatments. **(A)** Expression of immune factors (e.g. TGF-β, VCAM1, HMGB1, and FOXP3) in healthy controls. **(B)** Expression of immune factors in HFD intervention group. **(C)** Expression of immune factors in HFD with atorvastatin treated group. **(D)** Expression of immune factors in HFD with XFZY treated group. **(E)** Expression of immune factors in TMZY with atorvastatin treated group. **(F)** Expression of immune factors in HFD with the integrative treatment. **(G)** The bar plot indicating the digitalized intima-media thickness of thoracic aorta (which were generated by ipp6.0) of rats from six groups. The p values of significant associations are all marked on top of boxplots, while NS donates “non-significance”.

TMZY exhibited a strong and similar capability in regulating immunoregulatory factors induced by HFD. We examined the expression of immunoregulatory factors in the thoracic aorta of rats using IHC. The positive expression of immunoregulatory factors, indicated by brown-yellow to brown coloration, was located in the cytoplasm and nucleus of aortic endothelium and SMCs (**Figures 3A, B** **)**. Immunoreactive scores (IRS) (**Figure 3G**) showed that HFD disordered the expression of four immunoregulatory factors. TMZY intervention prominently up-regulated TGF-β expression (*P =* 0.038), and down-regulated VCAM-1 and HMGB-1 expression (*P =* 0.042 and *P =* 0.0021, respectively, **Figure 3**). No difference in the expression of four immunoregulatory factors was found between the TMZY-treated and XFZY-treated groups (*P =* 0.31 for TGF-β, *P =* 0.079 for VCAM-1, *P =* 0.06 for HMGB-1, and *P =* 0.061 for FOXP3). Interestingly, TMZY and atorvastatin also exhibited a similar effect on the expression of all immunoregulatory factors (*P =* 0.79 for TGF-β, *P =* 0.92 for VCAM-1, *P =* 0.25 for HMGB-1, and *P =* 0.22 for FOXP3). These suggested that TCM formulas and atorvastatin exhibited a highly similar effect on the immunoregulatory factors with each other.

**Figure 4:**
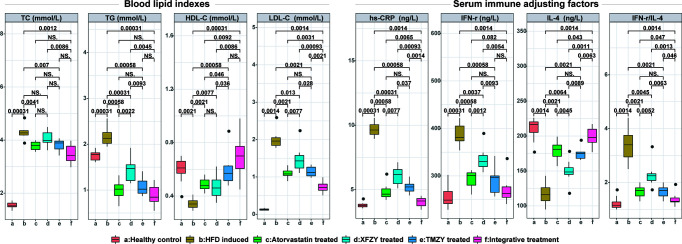
The blood lipid indexes and expression of serum immune factors in atherosclerotic rats were greatly improved under TCM treatments. Blood lipid indexes [i.e. TC (mmol/L), TG (mmol/L), HDL-C (mmol/L), and LDL-C (mmol/L)] and expression of serum immune factors [i.e. hs-CRP (ng/L), IFN-r (ng/L), and IL-4 (ng/L)] were respectively compared across all six groups. The p values of significant associations are all marked on top of boxplots, while NS donates “non-significance”.

We further found that TMZY balanced the HFD-derived disturbance in the four blood lipid indices (**Figure 4**). HFD dramatically elevated the levels of TC, TG, LDL-C (*P =* 0.0031, *P =* 0.0031, and *P =* 0.0014, respectively), while prominently lowering the level of HDL-C (*P =* 0.0021). TMZY intervention dramatically decreased the levels of TC, TG, and LDL-C (*P =* 0.007, *P =* 0.00058, and *P =* 0.0021, respectively) while prominently elevating HDL-C level (*P =* 0.00058), compared to the HFD group. The effects of TMZY on reducing TG level (*P =* 0.0093) and LDL-C (*P =* 0.028) level as well as elevating HDL-C level (*P =* 0.036) were significantly stronger than that of XFZY, while its effects on increasing HDL-C level were stronger than that of atorvastatin (*P =* 0.046).

TMZY exerted anti-inflammatory effects on the HFD rats. We first found distinct alterations in the four inflammatory cytokines due to HFD. The levels of high serum sensitivity C-reactive protein (hs-CRP) and the ratio of interferon (IFN)-γ/interleukin (IL)-4 were significantly elevated by HFD (*P =* 0.00031 and *P =* 0.0014, respectively). Afterward, TMZY intervention significantly lowered the hs-CRP level (*P =* 0.00058) and IFN-γ level (*P =* 0.00058), and elevated IL-4 level (*P =* 0.0021), thus reducing the ratio of IFN-γ/IL-4 (*P =* 0.0021) from the HFD group. Moreover, the regulatory effects of TMZY were more substantial than that of XFZY on hs-CRP (*P =* 0.037), IFN-γ (*P =* 0.0093), IL-4 (*P =* 0.0089), and IFN-γ/IL-4 (*P =* 0.0063) levels. No difference in all inflammatory cytokine levels was observed between the TMZY and atorvastatin intervention (*P =* 0.32 for hs-CRP, *P =* 0.41 for IFN-γ, *P =* 0.44 for IL-4, and *P =* 1 for IFN-γ/IL-4).

As a classic TCM formula, the regulatory capability of XFZY in the blood lipid levels and inflammatory cytokines still need improvement compared to atorvastatin. Firstly, the effect of XFZY on the lipid regulation (*P =* 0.0022 for TG level, and *P =* 0.0077 for LDL-C level) was smaller than that of atorvastatin. Secondly, the beneficial effect of XFZY on the inflammatory cytokines is not as significant as that of atorvastatin intervention (*P =* 0.0077 for hs-CRP; *P =* 0.0052 for IFN-γ/IL-4).

These results above indicated that the effects of TMZY intervention on reducing the thickness of aortic intima and lowing levels of TG, LDL-C, and inflammatory cytokines were superior to that of the classic TCM formula (XFZY). Furthermore, TMZY overall exhibited similar therapeutic effects on AS (i.e., pathological morphology changes, expression of aortic immunoregulatory factors, and levels of inflammatory cytokines) with atorvastatin, while only producing higher HDL-C level than atorvastatin.

### The Effect of a Combination of TMZY and Atorvastatin Is More Significant Than Any Individual Ones

Either TMZY or atorvastatin exerted a substantial effect on atherosclerotic rats. However, it is still not clear if TMZY and atorvastatin interacted in permissive, synergistic, or even antagonistic ways on AS. Here, we next assessed the combined effect of TMZY and atorvastatin by examining the aforementioned phenotypic changes in atherosclerotic rats across treatment groups.

We found that integrative treatment can significantly alleviate the pathological changes in the structure of arteria by HFD (**Figure 2F**). The aortic intimal hyperplasia was reduced with complete structure, and the arrangement of SMCs in intima was relatively regular. No sclerotic plaques in the integrative-treatment group were observed, the structure of each layer was clear, and the pathological changes by HFD were significantly reduced. In the pixel-based analysis, the integrative treatment effectively inhibited the formation of atherosclerotic plaques by decreasing the thickness of intima and media (*P =* 8.9e-06 and *P =* 7.8e-05, respectively; **Figures 2G, H** **)**. Such a protective role of the integrative treatment was significantly superior to both atorvastatin (*P =* 0.012 and *P =* 0.035, respectively) and XFZY (*P =* 0.00022 and *P =* 0.022, respectively). Notably, the integrative treatment was marginally superior to the TMZY (*P =* 0.05) on the reduction of media thickness, suggesting that TMZY may play a key role in reducing media thickness in the integrative treatment.

We next sought to identify how integrative treatment regulated the four immune factors. The integrative treatment prominently up-regulated TGF-β and FOXP3 expression (*P =* 0.028 and *P =* 0.0024), and down-regulated VCAM-1 and HMGB-1 expression (*P =* 5.2e-05 and *P =* 0.00024, respectively) induced by HFD (**Figures 3B, F, G** **)**. The protective effects of the integrative treatment on FOXP3 expression were superior to atorvastatin (*P =* 0.021) (**Figures 3C, F, G** **)**, while its effects on VCAM-1, HMGB-1 and FOXP3 expression were superior to XFZY (*P =* 8.9e-05, *P =* 0.0075, and *P =* 0.0026, respectively) (**Figures 3D, F, G** **)**. Remarkably, no difference was observed in the treatment effect on all immune factors between the TMZY and the integrative treatment groups (*P =* 0.95 for TGF-β, *P =* 0.11 for VCAM-1, *P =* 0.64 for HMGB-1, and *P =* 0.6 for FOXP3) (**Figures 3E–G**), suggesting that TMZY primarily contributed to the observed effect of the integrative treatment on the expression of related immune factors.

The integrative treatment further lowered the blood lipid levels. We first found that it considerably lowered the levels of TC, TG, and LDL-C (*P =* 0.0012, *P =* 0.00031, and *P =* 0.0014, respectively; **Figure 4**), while significantly elevated HDL-C level (*P =* 0.00031) induced by HFD. The integrative treatment reduced LDL-C level and elevated HDL-C level more than did atorvastatin (*P =* 0.0031 and *P =* 0.0092, respectively). Its protective effects on all blood lipid indices were notably more reliable than those of XFZY (*P =* 0.0086, *P =* 0.0045, *P =* 0.00093, and *P =* 0.0086, respectively), where it only exhibited a substantial effect on reducing LDL-C level than that of TMZY (*P =* 0.0021).

Moreover, the integrative treatment outperformed others in the regulation of immune-inflammatory cytokines. It significantly lowered hs-CRP level (*P =* 0.0014) and IFN-γ level (*P =* 0.0014), elevated IL-4 level (*P =* 0.0014), and a reduced ratio of IFN-γ/IL-4 (*P =* 0.0014) induced by HFD. Moreover, its regulatory effects were more substantial than that of atorvastatin on hs-CRP (*P =* 0.0065), IFN-γ (*P =* 0.082), IL-4 (*P =* 0.043), and IFN-γ/IL-4 (*P =* 0.047) levels. Similarly, the integrative treatment outperformed XFZY in regulating the level of hs-CRP (*P =* 0.00093), IFN-γ (*P =* 0.0054), IL-4 (*P =* 0.0011), and IFN-γ/IL-4 (*P =* 0.0013). Besides, its effects were also more reliable than that of TMZY on hs-CRP (*P =* 0.0014), IL-4 (*P =* 0.0063), and IFN-γ/IL-4 (*P =* 0.046) levels.

Taken together, the integrative treatment on rats exhibited a more significant protective effect on AS-associated pathological morphology, expression of host immune factors, and levels of blood lipid and inflammatory cytokines, providing novel insights into the development of novel therapeutic strategy on AS by adopting the innovative TMZY formula in combination with atorvastatin in the regular clinical settings.

### Effects of TMZY on Body Weight and Platelet Aggregation in HCD-Fed Mice

**Figure 5:**
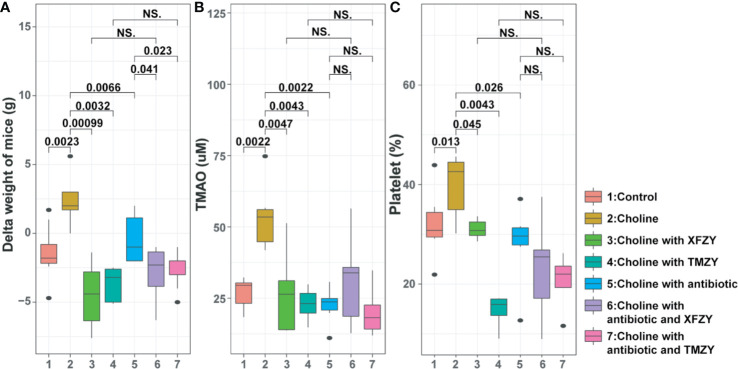
The phenotypic changes (weight-gain, concentration of TMAO, and platelet aggregation) of mice in the high-fat diet (HFD) by TCM treatments with or without the antibiotic pre-treatment. **(A)** Boxplots of weight-gain of mice from (1) control group, (2) HFD groups, (3) HFD with XFZY treatment, (4) HFD with TMZY treatment, (5) HFD with antibiotic intervention, (6) HFD with antibiotic intervention and XFZY treatment, (7) HFD with antibiotic intervention and TMZY treatment. **(B)** Boxplots of the TMAO level (μM) in mice among the seven groups. **(C)** Boxplots of the platelet aggregation (%) in mice among the seven groups.

To reveal the underlying mechanisms of actions of TCM formulas, we fed mice at HCD with the treatment of XFZY or TMZY. Firstly, the diet intervention with high-choline notably elevated body weight of mice (*P =* 0.0023, **Figure 5A**), TMAO level (*P =* 0.0022, **Figure 5B**), and platelet aggregation (*P =* 0.013, **Figure 5C**) compared with the control group. Then, we found that both XFZY and TMZY could prominently reduce body weight (*P =* 0.00099 and *P =* 0.0032, respectively), TMAO level (*P =* 0.0047 and *P =* 0.0043, respectively) and platelet aggregation (*P =* 0.045 and *P =* 0.0043, respectively). Results described above implied that both XFZY and TMZY could effectively reduce the concentration of TMAO and platelet in the blood of mice, and inhibit the risk of obesity caused by high-choline, suggesting a therapeutic effect of these two formulas on diseases such as hyperlipidemia.

### The Modified TCM Formula Functioned Through Gut Microbiota

To explore the potential role of gut microbiomes in the regulatory mechanism of TMZY, antibiotics treatments were used in our study. Administration of quadruple antibiotics notably lowered body weight (*P =* 0.0066), TMAO level (*P =* 0.0022), and platelet level (*P =* 0.026), compared with the choline group, suggesting these phenotypes are driven by microbial dysbiosis in the gut microbiota. Under the administration of quadruple antibiotic, both XFZY and TMZY still reduced body weight of mice slightly (*P* = 0.041, *P* = 0.023, **Figure 4A**) compared to the choline-plus-antibiotic group (**Figure 4A**). At the same time, the levels of TMAO (**Figure 4B**) and platelet remained unchanged (**Figure 4C**) after treatment with either XFZY or TMZY compared to the choline-plus-antibiotic group. Therefore, it suggested that TCM formulas probably improved atherosclerotic therapeutic outcomes by the structural modulation of gut microbiota of mice.

### TMZY Modulated the Structure and Function in the Gut Microbiota of Mice Under the HCD Diet

**Figure 6:**
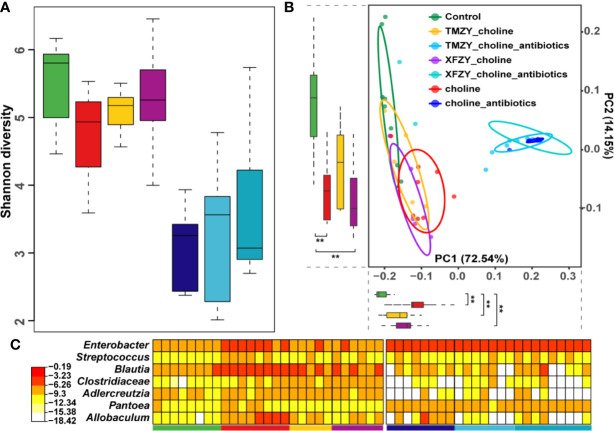
The dysbiosis in the gut microbiota of HFD mice was perturbed and rehabilitated by TCM treatments. **(A)** Boxplots of Shannon diversity among (1) controls, (2) HFD groups, (3) HFD with XFZY treatment, (4) HFD with TMZY treatment, (5) HFD with antibiotic intervention, (6) HFD with antibiotic intervention and XFZY treatment, (7) HFD with antibiotic intervention and TMZY treatment. **(B)** PCoA plot of fecal microbiota from the seven groups based on Meta-Strom distance. The boxplots of PC1 and PC2 were presented as well. Statistical significance is marked as ** (*p* < 0.005). **(C)** Heat-map of differentially abundant genera between HFD group and healthy controls, where the relative abundance of those marker genera in other five groups were also presented as reference. The color indicates the log10-scaled relative abundance of a bacterial genus.

To further explore how the TCM formula (TMZY) regulates AS by interacting with gut microbiota, microbiome analysis on fecal samples of mice was performed based on 16S rRNA sequencing. Firstly, gut microbiota in control diet exhibited the highest level of alpha diversity (Shannon index) among all groups, while those in the HCD showed the lowest level of alpha diversity among all groups without antibiotics treatments, suggesting choline diet may trigger intestinal microbial dysbiosis by reducing the bacterial alpha-diversity in the gut (*P =* 0.05, **Figure 6A**). After treatment with TMZY, we found that alpha-diversity was slightly improved compared to the HCD group. On the other hand, under the administration of antibiotics, the difference in alpha diversity among all groups was not significant. Next, beta-diversity was calculated for each sample to evaluate the similarity between microbial communities. A principal coordinate analysis (PCoA) was performed based on the UniFrac distance of the 16S rRNA sequence profiles at the OTU level (**Figure 6B**). Two primary clusters of fecal samples were formed by antibiotics administration along with principal component (PC) 1. In the non-antibiotics cluster, it was first found that intestinal microbiotas from the control and high-choline groups were highly distinct concerning organismal structure, with mice forming two major sub-clusters that corresponded to the two diet groups. Along the PC 2, the microbiota from mice underwent TCM treatments shifted from those of HCD toward a healthy structure revealed by mice in the control group, where TMZY-treated microbiotas were more similar to the healthy one compared to the XFZY-treated microbiota. Remarkably, the relative abundance of bacteria, such as *Enterobacter*, *Streptococcus*, *Blautia*, *Clostridiacea*, *Adlercreutzia*, *Pantoea*, and *Allobaculum*, had shown a strong resilience related to the treatment of TMZY on mice under HCD, which bounced back to the normal level after TCM treatments (**Figure 6C**). Moreover, the observed plastic patterns in microbiome structures were verified in the antibiotic-treated mice.

**Figure 7:**
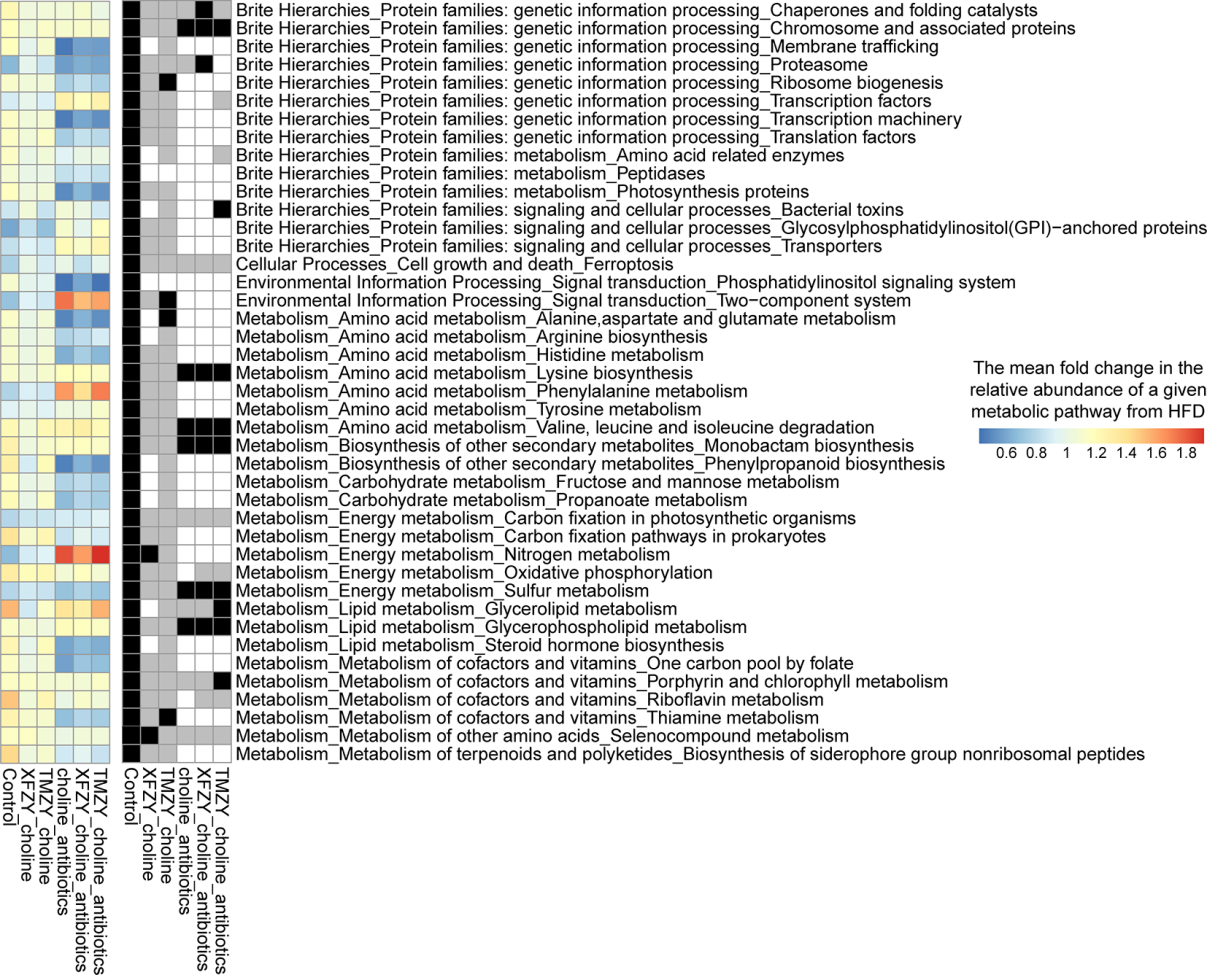
Microbial functional response in the gut microbiome to TCM treatments on HFD mice. In the left panel, a heatmap indicate the mean fold change of the relative abundance of a given pathway in a treatment group over the HFD group. All 42 pathways are significantly different between healthy controls from HFD group, which were designated as a “reference” microbial response to HDF that presumably corresponds to a full recovery in the gut microbiome from HFD (the leftmost column in the heatmap). The right panel showed the statistical significance (*p* value) of the fold changes of corresponding pathways in the left panel and its association directionality under a treatment as compared to HDF group. A black cell indicates a significant change in the corresponding pathway toward the healthy status under a treatment. A gray cell refers to a health-associated shift but no statistical significance was found. A white cell indicates neither a health-associated shift nor a statistical significance related to HFD was found in a pathway under a treatment.

To further build the links between compositions and functions of gut microbiome related to TCM treatments, PICRUST2 was employed to impute the microbial metabolic capacities based on the 16S data of gut microbiome (Gavin M. Douglas et al., 2019). All imputed functional profiles were summarized to the pathway level for downstream analysis. Firstly, we presented the functional dysbiosis in the gut microbiome during the HFD without any treatments. It was marked by characteristic shifts in functional profiles, including 42 critical metabolic pathways that distinguish healthy controls from HFD mice (**Figure 7**). It can also designate as a “reference” microbial response that presumably corresponds to a full recovery that a given treatment can promote from the HFD. Next, we presented the differential levels of microbial functional response to treatments compared to that reference microbial response to reveal how well a given treatment can change the dysbiosis states in the hosts by remodeling of gut microbial functions. TMZY induced the most similar microbial response in functional profiles of the gut microbiome to that in the “reference,” where 40 out of 42 metabolic pathways had the identical trend as the reference and four of them were significantly changed (e.g. Two-component system, Alanine, aspartate, and glutamate metabolism, Thiamine metabolism, and Ribosome biogenesis), while XFZY only have 30 characteristic pathways with identical change trend as the reference.

On the other hand, the mice under the antibiotics intervention harbored a remarkably different response in functional profiles of the gut microbiome from the reference response. Thus, the functional response in the gut microbiome was able to characterize the pharmacological effects of TCM formulas and could further leverage to the systematic comparisons of treatment effectiveness in the drug development. Taken together, microbial dysbiosis in the gut microbiota induced AS and CVD indirectly by causing chronic metabolic dysfunction, or producing proatherogenic molecules from dietary precursors, whereas TCM formulas were capable of reverting such dysbiosis by modulating the vital metabolic functions in the gut microbiome related to atherogenesis.

## Discussion

To our knowledge, this is the first study that systematically reported the pharmacological effects of both TCM formulas and Western medicine on treating AS in the animal model. TCM played an essential role in CVD disease treatment and prevention for thousands of years in China. Recent studies have reported that inflammation plays a critical role in the formation and development of AS (Lu and Kakkar, 2014). A variety of TCM compounds or monomers for activating blood have shown to inhibit the inflammatory response of AS (Kang et al., 2015; Zeng et al., 2018; Liu et al., 2020). To achieve the innovative development of TCM, there is an unmet need to improve the TCM pharmacological effects by modifying formulas with a better understanding of the underlying mechanism of TCM in the context of both host phenotypes and the gut microbiome.

XFZY is a typical TCM formula to treat AS in the ancient years. Wang Qingren first recorded it in a book named Yi Lin Gai Cuo. XFZY treatment was able to reduce blood lipids and regulate immune inflammation in atherosclerotic rats. XFZY is still acting as one of the most representative essential prescriptions for the clinical treatment of CVD at present (Shi et al., 2017). We thus compared the pharmacological effects of this classic TCM formulas on AS with that of Western medicine. Statins (such as atorvastatin) has been widely used as classic drugs to relieve symptoms in atherosclerotic rats (Rodriguez et al., 2017; Sun et al., 2018). However, the protective effects of XFZY on thoracic aortic intima thickness, for instance, the levels of TG, LDL-C, and inflammatory factors were slightly weaker than that of atorvastatin.

We next sought to test if the pharmacological effects of classic TCM formulas (such as XFZY) can be improved by adding several constituents according to TCM theory. To address this challenge, we first systematically studied the CVD-related TCM herbal compounds and searched for candidate components to modify XFZY. A massive number of individual compounds from TCM formulas documented have excellent therapeutic effects on atherosclerotic CVD, such as ligustrazine, safflower, notoginsenoside, and tanshinone. Many of them were reported to alleviate AS effectively by enhancing anti-platelet aggregation activity, protecting vascular endothelium, remodeling of myocardium, lowering blood lipid and improvement of microcirculation (Chen, 2015; Xu and Chen, 2017). We finally curated and included four TCM monomers in the TMZY formula [*Citrus aurantium* L. (Zhi Shi) (Li and Wang, 2011), *Crataegus pinnatifida* Bunge (Shan Zha) (Fan et al., 2006; Akila and Devaraj, 2008), *Reynoutria multiflora* (Thunb.) Moldenke (syn. *Fallopia multiflora* (Thunb.) Harald.) (Shou Wu) (Zhang et al., 2018), and *Alisma Plantago-aquatica* Linn. (Ze Xie) (Zhang et al., 2017)].

The TMZY (an optimized formula of XFZY) further enhanced the interventional effect on atherosclerotic rats. It offset the disadvantages of XFZY in reducing intima thickness, lowering levels of TG and LDL-C, and decreasing levels of inflammatory factors. Furthermore, we observed that TMZY and atorvastatin could probably synergistically attenuate aortic intima thickening and foam cell accumulation, downregulate the inflammation, blood lipid level, *etc.* The therapeutic effects of TMZY on atherosclerotic rats were highly similar to those of atorvastatin, while it further improved several immunological measurements which were less optimistic in the atorvastatin treatment.

The heart-gut axis has been a well-established role for the gut microbiome in the pathogenesis of CVD. Recently, it has been reported that the diversity of gut microbiome negatively correlated with AS (Menni et al., 2018). The metabolites of the gut microbiome, such as trimethylamine oxide, urotoxin, short-chain fatty acid, phytoestrogen, anthocyanin, bile acid, and lipopolysaccharide, can affect the process of AS by intervening the metabolism of carbohydrate, lipid and choline, oxidative stress, immune-inflammatory factor release, and other ways (Wang and Zhao, 2018). Among these metabolites in the gut microbiome, TMAO is still the most attractive to research community due to its strong association with the incidence of atherosclerotic CVD and gut microbiome (Koeth et al., 2013; Zhu et al., 2016; Boini et al., 2017; Schuett et al., 2017). Microbiome-targeted interventions (such as fecal microbiome transplantation, probiotics, and so on) have been actively developed recently for the prevention and treatment of AS. Here, we borrowed the ideas of TCM in the treatment of CVD and developed a novel TCM formula to successfully intervene the process of AS by regulating systemic TMAO levels.

In this study, we established the interactions between gut microbiota and the TCM formulas (XFZY and TMZY) in the treatment of atherosclerotic disease induced by HCD. Both XFZY and TMZY effectively reduced body weight, platelet aggregation rate, and TMAO in the HCD mice by modulating the compositions of gut microbiomes. However, after antibiotics suppression of gut microbiome in mice, XFZY and TMZY only reduced the body weight of mice whereas exerting no effect on the platelet aggregation rate and TMAO. These suggested that TMAO is a highly gut microbe-dependent metabolite generated from dietary choline, which further enhanced the platelet activation. The changes in the gut microbiota induced by TCM formulas greatly contributed to the improvement of the systematic TMAO level and platelet reactivity. Interestingly, TCM formulas can lower the body weight of mice under HCD independently of the gut microbiota. We further compared the alterations in gut microbiota by multiple treatments, revealing why TMZY outperformed others in alleviating AS mediated by the dietary choline.

In summary, we first demonstrated the enhanced pharmacological effect of a modified TCM formula (TMZY) on AS model (HFD) rats. Furthermore, TMZY and atorvastatin could act together for the alleviation of AS in rats by suppressing immune and inflammatory responses. Our study also provided the compelling evidence that alterations of gut microbiota induced by TCM formulas are associated with the anti-AS effects of TCM formulas in mice. The rational validation of the microbiome-mediated phenotypic modulation of AS by Chinese herbal formula are still needed in the human populations for the development of novel therapeutic strategies of CVD treatments.

## Data Availability Statement

The datasets presented in this study can be found in online repositories. The names of the repository/repositories and accession number(s) can be found below: https://www.ncbi.nlm.nih.gov/, PRJNA615890.

## Ethics Statement

The animal study was reviewed and approved by: The Ethics Committee of Qingdao University approved all the experimental procedures, and animals were treated according to the recommendations in the Guide for the Care and Use of Laboratory Animals of the National Institutes of Health.

## Author Contributions

WJ and TJ carried out the experiment, ZS and CM did the data analysis, FT conducted the sample collection, WJ, TJ, ZS, and SH wrote the manuscript, and SH and SY supervised the work. All authors contributed to the article and approved the submitted version.

## Funding

This work was supported by 2017 Qingdao science and technology benefiting people special project (17-3-3-3-nsh), Qingdao Medical and health excellent talents training project (No. ZYYRC1701001; No. ZYYRC1702002; No.ZYYRC2002001), and National Science Foundation of China (No. 31800088); as well as Qingdao traditional Chinese medicine hospital (Qingdao Haici medical group) talent training project (hcr012019001, hcr032019012).

## Conflict of Interest

The authors declare that the research was conducted in the absence of any commercial or financial relationships that could be construed as a potential conflict of interest.

## Supplementary Material

Supplementary Table 1 HPLC peak assignment of TMZY extracts.

Supplementary Table 2 Abbreviations used in the paper.

## References

[B1] Akila M. Devaraj H. (2008). Synergistic effect of tincture of Crataegus and Mangifera indica L. extract on hyperlipidemic and antioxidant status in atherogenic rats. Vasc. Pharmacol. 49, 173–177. 10.1016/j.vph.2008.07.007 18755296

[B2] Benjamin E. J. Virani S. S. Callaway C. W. Chamberlain A. M. Chang A. R. Cheng S. (2018). Heart Disease and Stroke Statistics-2018 Update A Report From the American Heart Association. Circulation 137, E67–E492. 10.1161/CIR.0000000000000558 29386200

[B3] Boini K. M. Hussain T. Li P. L. Koka S. (2017). Trimethylamine-N-Oxide Instigates NLRP3 Inflammasome Activation and Endothelial Dysfunction. Cell Physiol. Biochem. 44, 152–162. 10.1159/000484623 29130962 PMC5828122

[B4] Chen K. J. (2015). Development track of the modern activating blood circulation and removing stasis (ABCRS) school on inheritance and innovation. Chin. J. Integr. Med. 21, 883–886. 10.1007/s11655-015-2092-7 26631172

[B5] Fan C. L. Yan J. Qian Y. Wo X. D. Gao L. P. (2006). Regulation of lipoprotein lipase expression by effect of hawthorn flavonoids on peroxisome proliferator response element pathway. J. Pharmacol. Sci. 100, 51–58. 10.1254/jphs.FP0050748 16404131

[B6] Douglas G. M. Maffei V. J. Zaneveld J. Yurgel S. N. Brown J. R. Taylor C. M. (2019). PICRUSt2: An improved and extensible approach for metagenome inference. bioRxiv. 10.1101/672295

[B7] Hao P. Jiang F. Cheng J. Ma L. Zhang Y. Zhao Y. (2017). Traditional Chinese Medicine for Cardiovascular Disease: Evidence and Potential Mechanisms. J. Am. Coll. Cardiol. 69, 2952–2966. 10.1016/j.jacc.2017.04.041 28619197

[B8] Ji W. Y. Jiang T. Liu Y. H. Ji Z. Q. (2017). Effect of New Xuefuzhuyu Soft Capsule on T Cell Subset Ratio of The Patients with Coronary Heart Disease Angina. J. Basic Chin. Med. 23, 1580–1582.

[B9] Ji W. Y. Zhao K. Chi W. F. Lin L. H. Yu Y. G. Ji Q. Z. (2012). Efect of New Xuefu Zhuyu Soft Capsule on the Blod Stasis Syndrome and Vascular Endothelial Inflammation in Patients with Coronary Heart Disease. J. Tradit. Chin. Med. 53, 1934–1936. 10.13288/j.11-2166/r.2012.22.010

[B10] Jie Z. Xia H. Zhong S. L. Feng Q. Li S. Liang S. (2017). The gut microbiome in atherosclerotic cardiovascular disease. Nat. Commun. 8, 845. 10.1038/s41467-017-00900-1 29018189 PMC5635030

[B11] Jing G. C. Sun Z. Wang H. L. Gong Y. H. Huang S. Ning K. (2017). Parallel-META 3: Comprehensive taxonomical and functional analysis platform for efficient comparison of microbial communities. Sci. Rep. 7, 40371. 10.1038/srep40371 28079128 PMC5227994

[B12] Jonsson A. L. Backhed F. (2017). Role of gut microbiota in atherosclerosis. Nat. Rev. Cardiol. 14, 79–87. 10.1038/nrcardio.2016.183 27905479

[B13] Kang Q. Liu W. Liu H. Zhou M. (2015). Effect of Compound Chuanxiong Capsule on Inflammatory Reaction and PI3K/Akt/NF-kappaB Signaling Pathway in Atherosclerosis. Evid. Based. Complement Alternat. Med. 2015, 584596. 10.1155/2015/584596 26539229 PMC4619937

[B14] Koeth R. A. Wang Z. Levison B. S. Buffa J. A. Org E. Sheehy B. T. (2013). Intestinal microbiota metabolism of L-carnitine, a nutrient in red meat, promotes atherosclerosis. Nat. Med. 19, 576–585. 10.1038/nm.3145 23563705 PMC3650111

[B15] Komaroff A. L. (2018). The Microbiome and Risk for Atherosclerosis. JAMA 319, 2381–2382. 10.1001/jama.2018.5240 29800043

[B16] Koren O. Spor A. Felin J. Fak F. Stombaugh J. Tremaroli V. (2011). Human oral, gut, and plaque microbiota in patients with atherosclerosis. Proc. Natl. Acad. Sci. U. S. A. 108 (Suppl 1), 4592–4598. 10.1073/pnas.1011383107 20937873 PMC3063583

[B17] Li C. Wang M. H. (2011). Anti-inflammatory effect of the water fraction from hawthorn fruit on LPS-stimulated RAW 264.7 cells. Nutr. Res. Pract. 5, 101–106. 10.4162/nrp.2011.5.2.101 21556222 PMC3085797

[B18] Lin F. Chen B. L. Wang Y. Z. Gao D. Song J. Kaptchuk T. J. (2018). In Vitro Angiogenesis Effect of Xuefu Zhuyu Decoction () and Vascular Endothelial Growth Factor: A Comparison Study. Chin. J. Integr. Med. 24, 606–612. 10.1007/s11655-015-2289-9 26272550 PMC4779078

[B19] Liu B. Song Z. Yu J. Li P. Tang Y. Ge J. (2020). The atherosclerosis-ameliorating effects and molecular mechanisms of BuYangHuanWu decoction. BioMed. Pharmacother. 123, 109664. 10.1016/j.biopha.2019.109664 31887542

[B20] Liu C. Huang Y. (2016). Chinese Herbal Medicine on Cardiovascular Diseases and the Mechanisms of Action. Front. Pharmacol. 7, 469. 10.3389/fphar.2016.00469 27990122 PMC5130975

[B21] Liu H. Wang S. Sun A. Huang D. Wang W. Zhang C. (2012). Danhong inhibits oxidized low-density lipoprotein-induced immune maturation of dentritic cells via a peroxisome proliferator activated receptor gamma-mediated pathway. J. Pharmacol. Sci. 119, 1–9. 10.1254/jphs.11226FP 22739234

[B22] Lu X. Kakkar V. (2014). Inflammasome and atherogenesis. Curr. Pharm. Des. 20, 108–124. 10.2174/13816128113199990586 23944376

[B23] Marchesi J. R. Adams D. H. Fava F. Hermes G. D. Hirschfield G. M. Hold G. (2016). The gut microbiota and host health: a new clinical frontier. Gut 65, 330–339. 10.1136/gutjnl-2015-309990 26338727 PMC4752653

[B24] Mcdonald D. Price M. N. Goodrich J. Nawrocki E. P. Desantis T. Z. Probst A. (2012). An improved Greengenes taxonomy with explicit ranks for ecological and evolutionary analyses of bacteria and archaea. Isme J. 6, 610–618. 10.1038/ismej.2011.139 22134646 PMC3280142

[B25] Meng F. Lai H. Luo Z. Liu Y. Huang X. Chen J. (2018). Effect of Xuefu Zhuyu Decoction Pretreatment on Myocardium in Sepsis Rats. Evid. Based. Complement Alternat. Med. 2018, 2939307. 10.1155/2018/2939307 30271451 PMC6151246

[B26] Menni C. Lin C. Cecelja M. Mangino M. Matey-Hernandez M. L. Keehn L. (2018). Gut microbial diversity is associated with lower arterial stiffness in women. Eur. Heart J. 39, 2390–2397. 10.1093/eurheartj/ehy226 29750272 PMC6030944

[B27] Methe B. A. Nelson K. E. Pop M. Creasy H. H. Giglio M. G. Huttenhower C. (2012). A framework for human microbiome research. Nature 486, 215–221. 10.1038/nature11209 22699610 PMC3377744

[B28] Reikvam D. H. Erofeev A. Sandvik A. Grcic V. Jahnsen F. L. Gaustad P. (2011). Depletion of murine intestinal microbiota: effects on gut mucosa and epithelial gene expression. PLoS One 6, e17996. 10.1371/journal.pone.0017996 21445311 PMC3061881

[B29] Rodriguez F. Maron D. J. Knowles J. W. Virani S. S. Lin S. Heidenreich P. A. (2017). Association Between Intensity of Statin Therapy and Mortality in Patients With Atherosclerotic Cardiovascular Disease. JAMA Cardiol. 2, 47–54. 10.1001/jamacardio.2016.4052 27829091

[B30] Romano K. A. Dill-Mcfarland K. A. Kasahara K. Kerby R. L. Vivas E. I. Amador-Noguez D. (2018). Fecal Aliquot Straw Technique (FAST) allows for easy and reproducible subsampling: assessing interpersonal variation in trimethylamine-N-oxide (TMAO) accumulation. Microbiome 6, 91–91. 10.1186/s40168-018-0458-8 29776435 PMC5960144

[B31] Schuett K. Kleber M. E. Scharnagl H. Lorkowski S. Marz W. Niessner A. (2017). Trimethylamine-N-oxide and Heart Failure With Reduced Versus Preserved Ejection Fraction. J. Am. Coll. Cardiol. 70, 3202–3204. 10.1016/j.jacc.2017.10.064 29268932

[B32] Shi X. Zhu H. Zhang Y. Zhou M. Tang D. Zhang H. (2017). XuefuZhuyu decoction protected cardiomyocytes against hypoxia/reoxygenation injury by inhibiting autophagy. BMC Complement Altern. Med. 17, 325. 10.1186/s12906-017-1822-0 28629357 PMC5477241

[B33] Stone N. J. Robinson J. G. Lichtenstein A. H. (2015). 2013 ACC/AHA Guideline on the Treatment of Blood Cholesterol to Reduce Atherosclerotic Cardiovascular Risk in Adults: A Report of the American College of Cardiology/American Heart Association Task Force on Practice Guidelines (vol 63, pg 2889, 2014). J. Am. Coll. Cardiol. 66, 2812–2812. 10.1161/01.cir.0000437738.63853.7a 24239923

[B34] Su X. Q. Xu J. Ning K. (2012). Meta-Storms: efficient search for similar microbial communities based on a novel indexing scheme and similarity score for metagenomic data. Bioinformatics 28, 2493–2501. 10.1093/bioinformatics/bts470 22843983

[B35] Sun B. Rui R. Pan H. Zhang L. Wang X. (2018). Effect of Combined Use of Astragaloside IV (AsIV) and Atorvastatin (AV) on Expression of PPAR-gamma and Inflammation-Associated Cytokines in Atherosclerosis Rats. Med. Sci. Monit. 24, 6229–6236. 10.12659/MSM.908480 30190450 PMC6139110

[B36] Tong X. Xu J. Lian F. Yu X. Zhao Y. Xu L. (2018). Structural Alteration of Gut Microbiota during the Amelioration of Human Type 2 Diabetes with Hyperlipidemia by Metformin and a Traditional Chinese Herbal Formula: a Multicenter, Randomized, Open Label Clinical Trial. MBio 9. 10.1128/mBio.02392-17 PMC5964358 29789365

[B37] Wang C. Niimi M. Watanabe T. Wang Y. Liang J. Fan J. (2018). Treatment of atherosclerosis by traditional Chinese medicine: Questions and quandaries. Atherosclerosis 277, 136–144. 10.1016/j.atherosclerosis.2018.08.039 30212682

[B38] Wang Z. Levison B. S. Hazen J. E. Donahue L. Li X. M. Hazen S. L. (2014). Measurement of trimethylamine-N-oxide by stable isotope dilution liquid chromatography tandem mass spectrometry. Anal. Biochem. 455, 35–40. 10.1016/j.ab.2014.03.016 24704102 PMC4167037

[B39] Wang Z. Zhao Y. (2018). Gut microbiota derived metabolites in cardiovascular health and disease. Protein Cell 9, 416–431. 10.1007/s13238-018-0549-0 29725935 PMC5960473

[B40] Wei X. Tao J. Xiao S. Jiang S. Shang E. Zhu Z. (2018). Xiexin Tang improves the symptom of type 2 diabetic rats by modulation of the gut microbiota. Sci. Rep. 8, 3685. 10.1038/s41598-018-22094-2 29487347 PMC5829262

[B41] Wu T. R. Lin C. S. Chang C. J. Lin T. L. Martel J. Ko Y. F. (2019). Gut commensal Parabacteroides goldsteinii plays a predominant role in the anti-obesity effects of polysaccharides isolated from Hirsutella sinensis. Gut 68, 248–262. 10.1136/gutjnl-2017-315458 30007918

[B42] Xu H. Chen K. J. (2017). Practical diagnostic criterion of blood stasis syndrome : Activating blood circulation committee of Chinese association of integrative medicine. Chin. J. Integr. Med. 23, 243–244. 10.1007/s11655-016-2400-x 27921194

[B43] Xu J. Chen H. B. Li S. L. (2017). Understanding the Molecular Mechanisms of the Interplay Between Herbal Medicines and Gut Microbiota. Med. Res. Rev. 37, 1140–1185. 10.1002/med.21431 28052344

[B44] Xu J. Lian F. M. Zhao L. H. Zhao Y. F. Chen X. Y. Zhang X. (2015). Structural modulation of gut microbiota during alleviation of type 2 diabetes with a Chinese herbal formula. Isme J. 9, 552–562. 10.1038/ismej.2014.177 25279787 PMC4331591

[B45] Yang J. W. Guan W. J. Hu P. (2017). Xuefu Zhuyu decoction induces the proliferation, migration, adhesion of human umbilical vein endothelial cells via VEGF-VEGFR2 pathway. Biomed. Res. India 28, 3869–3873.

[B46] Zeng Y. Guan M. Li C. Xu L. Zheng Z. Li J. (2018). Bitter melon (Momordica charantia) attenuates atherosclerosis in apo-E knock-out mice possibly through reducing triglyceride and anti-inflammation. Lipids Health Dis. 17, 251. 10.1186/s12944-018-0896-0 30400958 PMC6220495

[B47] Zhang L. L. Xu W. Xu Y. L. Chen X. P. Huang M. Q. Lu J. J. (2017). Therapeutic potential of Rhizoma Alismatis: a review on ethnomedicinal application, phytochemistry, pharmacology, and toxicology. Ann. N. Y. Acad. Sci. 1401, 90–101. 10.1111/nyas.13381 28662316

[B48] Zhang M. Liu Y. Xu M. Zhang L. Liu Y. Liu X. (2019). Carotid artery plaque intervention with Tongxinluo capsule (CAPITAL): A multicenter randomized double-blind parallel-group placebo-controlled study. Sci. Rep. 9, 4545. 10.1038/s41598-019-41118-z 30872737 PMC6418108

[B49] Zhang Q. Xu Y. Lv J. J. Cheng M. X. Wu Y. Cao K. (2018). Structure characterization of two functional polysaccharides from Polygonum multiflorum and its immunomodulatory. Int. J. Biol. Macromol. 113, 195–204. 10.1016/j.ijbiomac.2018.02.064 29471090

[B50] Zhu W. Gregory J. C. Org E. Buffa J. A. Gupta N. Wang Z. (2016). Gut Microbial Metabolite TMAO Enhances Platelet Hyperreactivity and Thrombosis Risk. Cell 165, 111–124. 10.1016/j.cell.2016.02.011 26972052 PMC4862743

