# Supplementary material for: The Enhanced Pharmacological Effects of Modified Traditional Chinese Medicine in Attenuation of Atherosclerosis Is Driven by Modulation of Gut Microbiota
Source: Front Pharmacol. 2020 Oct 15;11:546589. doi: 10.3389/fphar.2020.546589 (PMC7593568; doi:10.3389/fphar.2020.546589)
Supplement: Supplementary Table 1 — HPLC peak assignment of TMZY extracts. [file Table_1.docx]

**Table S1**. HPLC peak assignment of TMZY extracts.

| **Peak No.** | **RT/min** | **Component** | **Peak assignment to herbs** |
| --- | --- | --- | --- |
| 1 | 6.09 | ursolic acid | Polygonum multiflorum |
| 2 | 8.69 | Not identified | Hawthorn; Rhizoma alismatis |
| 3 | 13.02 | Not identified | Polygonum multiflorum; Radix rehmanniae; Rhizoma alismatis |
| 4 | 17.54 | Not identified | Achyranthesbidentata |
| 5 | 18.67 | Not identified | Radix paeoniae rubra |
| 6 | 19.46 | hydroxyl-safflor yellow A | Flos carthami |
| 7 | 20.09 | Not identified | Peach kernel; Flos carthami |
| 8 | 20.85 | Not identified | Angelica sinensis; Rhizoma Chuanxiong; Hawthorn; Citrus aurantium |
| 9 | 21.94 | Not identified | Citrus aurantium |
| 10 | 23.90 | Not identified | unknown |
| 11 | 25.07 | paeoniflorin | Radix paeoniae rubra |
| 12 | 30.01 | ferulic acid | Angelica sinensis; Rhizoma Chuanxiong |
| 13 | 35.83 | Not identified | Citrus aurantium |
| 14 | 41.49 | Not identified | Citrus aurantium |
| 15 | 42.67 | Not identified | unknown |
| 16 | 43.97 | naringin | Citrus aurantium |
| 17 | 45.63 | hesperdin | Citrus aurantium |
| 18 | 48.44 | neohesperidin | Citrus aurantium |
