# Supplementary material for: The Enhanced Pharmacological Effects of Modified Traditional Chinese Medicine in Attenuation of Atherosclerosis Is Driven by Modulation of Gut Microbiota
Source: Front Pharmacol. 2020 Oct 15;11:546589. doi: 10.3389/fphar.2020.546589 (PMC7593568; doi:10.3389/fphar.2020.546589)
Supplement: Supplementary Table 2 — Abbreviations used in the paper. [file Table_2.docx]

**Table S2.** Abbreviations used in the paper.

| **Abbreviation** | **Full name** |
| --- | --- |
| **CVD** | Cardiovascular Diseases |
| **AS** | Atherosclerosis |
| **TCM** | Traditional Chinese Medicine |
| **XFZY** | Xuefu Zhuyu Decoction |
| **TMZY** | Tongmai Zhuyu Decoction |
| **ICWM** | Integrated Chinese And Western Medicine |
| **HCD** | High-Choline Diet |
| **TMAO** | Trimethylamine-N-Oxide |
| **HFD** | High-Fat Diet |
| **SMCs** | Smooth Muscle Cells |
| **IHC** | Immunohistochemistry |
| **IRS** | Immunoreactive Scores |
| **TC** | Total Cholesterol |
| **TG** | Triglyceride |
| **LDL-C** | Low-Density Lipoprotein Cholesterol |
| **HDL-C** | High-Density Lipoprotein Cholesterol |
| **TGF-β** | Transforming Growth Factor-Beta |
| **VCAM-1** | Vascular Vell Adhesion Molecule-1 |
| **HMGB-1** | High Mobility Group Chromosomal Protein B1 |
| **Foxp3** | Forkhead Box P3 |
| **HCD** | High Choline Diet |
| **hs-CRP** | High Sensitivity C-Reactive Protein |
| **IFN-γ** | Interferon-Gamma |
| **IL-4** | Interleukin-4 |
| **SPF** | Specific-Pathogen-Free |
| **SI** | Staining Intensity |
| **MRM** | Multiple-Reaction Monitoring |
| **ACD** | Acidic Citric Acid Glucose |
| **FSC** | Forward Scattered Light |
| **SSC** | Side Scattered Light |
| **OTUs** | Operational Taxonomic Units |
